# Supplementary material for: FRETpredict: a Python package for FRET efficiency predictions using rotamer libraries
Source: Commun Biol. 2024 Mar 9;7:298. doi: 10.1038/s42003-024-05910-6 (PMC10925062; doi:10.1038/s42003-024-05910-6)
Supplement: Supplementary file 2 — Supplementary Information [file 42003_2024_5910_MOESM2_ESM.pdf]

Supporting Information

FRETpredict: A Python package for FRET efficiency predictions using rotamer libraries

Daniele Montepietra[1,2,◕] Giulio Tesei[3,◕], João M. Martins[3], Micha B. A. Kunze[3], Robert B. Best[4] & Kresten Lindorff-Larsen[3,*]

[1] Department of Department of Chemical, Life and Environmental sustainability sciences, University of Parma, Parma 43125, Italy, [2] Istituto Nanoscienze – CNR-NANO, Center S3, via G. Campi 213/A, 41125 Modena, Italy, [3] Structural Biology and NMR Laboratory & the Linderstrøm-Lang Centre for Protein Science, Department of Biology, University of Copenhagen, Copenhagen, DK-2200, Denmark, [4] Laboratory of Chemical Physics, National Institute of Diabetes and Digestive and Kidney Diseases, National Institutes of Health, Bethesda, MD, 20892-0520, USA

◕These authors contributed equally to this work.

* robert.best2@nih.gov, lindorff@bio.ku.dk

Supplementary Notes

Supplementary Note 1:

Detailed description of the steps used to create new rotamer libraries.

1. *Generation of the conformational ensemble of the FRET probe.* We generated conformational ensembles of the FRET probes by performing replica exchange MD (REMD) simulations, using the force fields developed by Graen *et al.*[1] with some minor corrections[2]. From these trajectories, we here saved and analysed approximately 28,000 frames.

2. *Selection of the peaks of the distributions of dihedral angles in the linkers.* We calculated the distributions of the dihedral angles in the linker using the conformational ensembles from REMD as input. Combinations of the dihedral angles corresponding to peaks in the dihedral distributions were combined to generate distinct probe conformers corresponding to C1 cluster centers.

3. *First clustering step.* Trajectory frames are assigned to the C1 cluster centers of least-squares deviation of the dihedral angles.

4. *Second clustering step.* Averages over the dihedral angles in the trajectory frames assigned to each cluster center are calculated to generate a new set of C2 cluster centers. As the C2 cluster centers do not necessarily represent physical conformations of the probe, they cannot be directly used to build the rotamer library. Instead, the probe conformation with the minimum least-squares deviation from the C2 cluster center is chosen as the representative conformation of each center. Moreover, each C2 cluster center is assigned a weight equal to the number of conformations in the cluster (cluster population). When normalized over all clusters, this statistical weight approximates the Boltzmann probability of the representative conformation for a free dye in solution, p_i^{int}. These steps are sufficient for short linkers with few dihedral angles. However, for longer linkers extra steps are needed to decrease the number of rotamers while ensuring a good coverage of the conformational space.

5. *Filtering based on cluster populations.* In most cases, including all the C2 cluster centers into the rotamer library (e.g., 8776 conformers for Lumiprobe Cy7.5 L1R) would defeat the purpose of using the RLA as its computational cost would be considerable, albeit much lower than for an MD simulation with explicit probes. Therefore, we implemented a weight-based cutoff to reduce the number of conformations in the library while maintaining a balanced coverage of the conformational space sampled by the probes. Namely, we filtered out C2 clusters with fewer than 10, 20, or 30 members, thus obtaining new sets of C3 clusters, which will be referred in this work as large, medium, and small rotamer libraries, respectively. Since filtering by the assigned weights skews the remaining weights from the underlying Boltzmann distribution, we implemented a third clustering step, in which the conformations previously belonging to a discarded C2 cluster are moved to the C3 cluster of minimum least-squares deviation, and the p_i^{int} values are updated accordingly.

6. *Alignment and writing data to file.* The C3 cluster centers are aligned to the plane defined by the Cα atom and the C–N peptide bond. The resulting rotamer library is composed of a structure file (PDB format) and a trajectory file (DCD format) for the aligned FRET probe rotamers, and a text file containing the intrinsic Boltzmann weights of each rotamer state p_i^{int}.

Supplementary Note 2:

Detailed description of the rotamer library placement and weighting steps.

Rotamer library placement. The first step in calculating FRET efficiencies is to place the FRET probes from the rotamer library at the protein site to be labeled, following the same procedure introduced in DEER-PREdict[3]. Briefly, the fluorophore library coordinates are translated and rotated based on the positions of the backbone Cα, amide N, and carbonyl C atoms. This results in a perfect overlap with the N and Cα coordinates of the protein backbone and an approximate alignment with the carbonyl C, which ensures that the Cα–Cβ vector of the probe has the correct orientation relative to the side chain of the labeled residue.

Rotamer library weighting. For each protein conformation, the overall probability of the ith rotamer of a probe is estimated by combining the intrinsic and the external Boltzmann probabilities of the inserted probe, independently from the other probe. The intrinsic probabilities, p_i^{in}, are obtained from the clustering procedure performed on the representative dihedral conformations of the free dye in solution and are related to the free energy of the rotamer, ϵ_i^{int}, via Boltzmann inversion. Following the approach of Polyhach *et al.*[4], we account for the environment surrounding the FRET probe and calculate the probe-protein interaction energy, e_i^{ext}. This is achieved by summing up 12-6 Lennard-Jones pair-wise interaction energies between the heavy atoms of the probe and the surrounding protein within a 1-nm radius. The Lennard-Jones atomic radii (σ) and potential-well depth (ϵ) parameters are obtained from the CHARMM36m force field[5]. The σ parameters can be scaled by a "forgive" factor which is set through the input parameter `sigma_scaling` and defaults to 0.5. This scaling compensates for inaccuracies in the placement of the bulky FRET probe, which tend to lead to clashes even for conformers with reasonably correct orientations of the probe with respect to the side chain of the labeled residue. The contribution of electrostatic interactions between charged probe and protein atoms is also taken into account using a dielectric constant of 78, and can be turned off by setting the `electrostatic` input parameter to `False`. Hence, the overall probability of the ith rotamer state attached to the sth protein conformation is calculated as

$$p_{si} = p_i^{\text{int}} p_{si}^{\text{ext}} = p_i^{\text{int}} \frac{\exp(-e_{si}^{\text{ext}}/kT)}{Z_s}, \tag{S1}$$

where $Z_s = \sum_i p_i^{\text{int}} \exp(-e_{si}^{\text{ext}}/kT)$ is the partition function quantifying the fit of the rotamer in the embedding protein conformation. Low Z_s values result from large probe-protein interaction energies, suggesting tight placement of the probe due to either (i) misplacement of the rotamers or (ii) protein conformations incompatible with the presence of the FRET probe at the labeled site. Therefore, frames with $Z_s < 0.05$ are discarded in the FRET efficiency calculation to preclude spurious conformers from contributing to the ensemble average, corresponding to a situation in which all of the rotamers have a positive steric energy. In FRETpredict, the default Z_s cutoff can be conveniently replaced by a user-provided value.

Supplementary Note 3:

Detailed description of the reweighting approach based on protein-dye interactions

In the scheme described above in S2 Text, after discarding frames with steric clashes ($Z < 0.05$), the remaining frames are assigned equal weights. Conversely, in the reweighting approach, all frames are included in the FRET efficiency calculation. The contribution of each frame is weighted by dye-protein interaction energies so that steric clashes lead to down-weighting whereas negative energies result in larger weights. The per-frame weights w_s are obtained as

$$w_s = \frac{Z_{s,donor} \times Z_{s,acceptor}}{\sum_s Z_{s,donor} \times Z_{s,acceptor}}, \tag{S2}$$

where $Z_{s,donor}$ and $Z_{s,acceptor}$ are the Boltzmann partition functions of the donor and the acceptor in the s-th frame. The weights are normalized so that $\sum_s w_s = 1$ and the effective fraction of frames contributing to the reweighted ensemble can be computed as $\phi_{eff} = \exp(S)$ where $S = -\sum_s w_s \cdot \ln(w_s)$.

Supplementary Figures and Tables

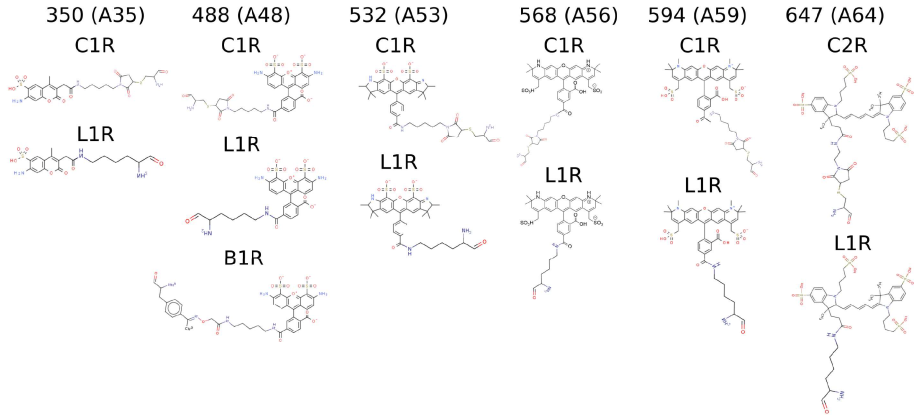

Supplementary Figure 1: **Structural formulae of the 13 AlexaFluor probes for which we generated rotamer libraries.** Each column corresponds to a different fluorophore (acronym in parentheses). The names of the linkers are reported above each formula.

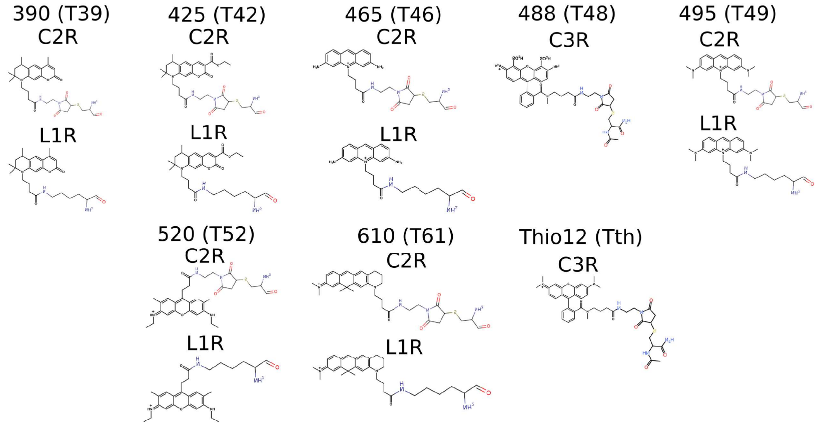

Supplementary Figure 2: **Structural formulae of the 14 ATTO probes for which we generated rotamer libraries.** Each column corresponds to a different fluorophore (acronym in parentheses). The names of the linkers are reported above each formula.

	large	medium	small
Donor clusters	706	124	32
Acceptor clusters	574	106	38
Computation time	692 s	120 s	37 s

Supplementary Table 1: **Computational times obtained using different cutoffs.** Computational times required to calculate FRET efficiencies from a pp11 trajectory of 316 frames (Case study 1) using the large (cutoff = 10), medium (cutoff = 20), and small rotamer libraries for AlexaFluor 488 - C1R and AlexaFluor 594 - C1R, on a laptop with AMD Ryzen 7 4800h processor with a Radeon graphics card. Compared to the large library, the medium library has significantly fewer cluster centers and it lowers the computational cost by a factor 6. Instead, choosing the small over the medium rotamer library results in a gain in computation time of around a factor of 3.

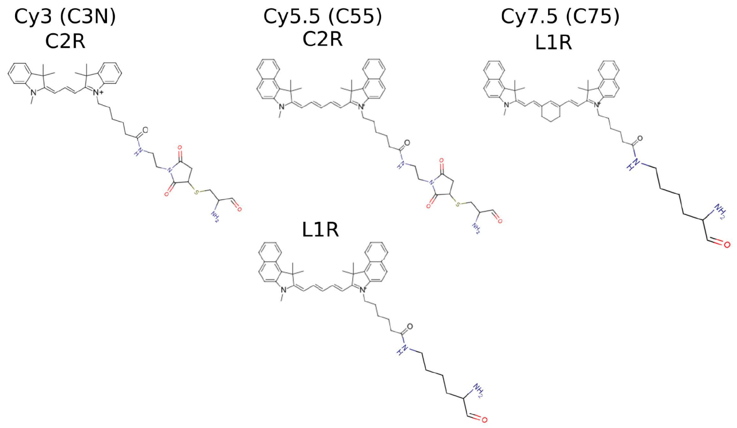

Supplementary Figure 3: **Structural formulae of the four Lumiprobe probes for which we generated rotamer libraries.** Each column corresponds to a different fluorophore (acronym in parentheses). The names of the linkers are reported above each formula.

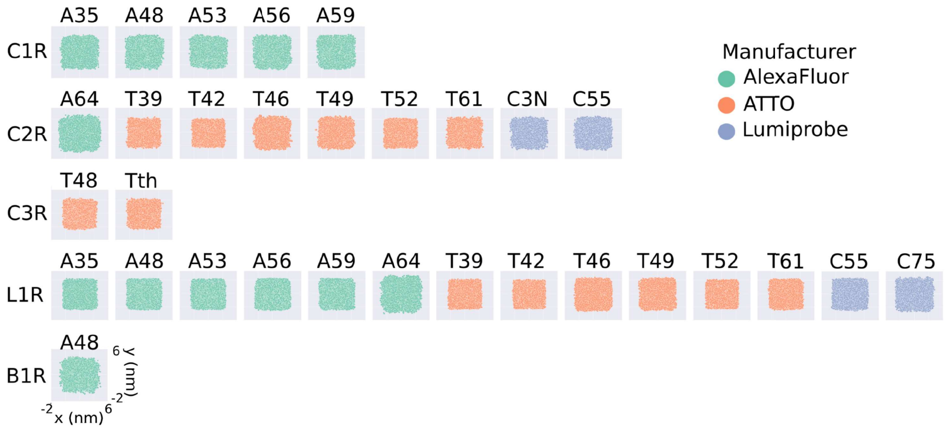

Supplementary Figure 4: **2D projections of the position of the fluorophore's central atom with respect to the Cα atom for the unfiltered rotamer libraries (cutoff = 0) generated in this work (C2 cluster centers).** The projections are obtained as the x and y coordinates of the central atom of the fluorophore (*O91* for AlexaFluor, *C7* for ATTO, and *C10* for Lumiprobe), after placing the Cα atom at the origin. Each plot represents a different FRET probe, divided into rows according to linker type (C1R, C2R, C3R, L1R, B1R, from top to bottom), and colored according to the manufacturer (green for AlexaFluor, orange for ATTO, and blue for Lumiprobe).

Polyproline 11 (pp11)			
Regime	small	medium	large
Static	0.732	0.745	0.743
Dynamic	0.876	0.886	0.881
Dynamic+	0.993	0.972	0.853
Average	0.917	0.912	0.89

Supplementary Table 2: **FRETpredict E for Case study 1: pp11.** FRET efficiencies calculated for pp11 using FRETpredict with different rotamer library sizes and three averaging regimes (static, dynamic, dynamic+) as well as the average over those. The reference experimental value is 0.88 whereas the value obtained as the average over the three regimes from MD simulations with explicit FRET probes is 0.83.

ACTR			
Residue pair (Regime)	[Urea] = 0 M	[Urea] = 2.5 M	[Urea] = 5 M
3-61 (Exp)	0.610	0.490	0.420
3-61 (Static)	0.602	0.374	0.319
3-61 (Dynamic)	0.698	0.451	0.382
3-61 (Dynamic+)	0.763	0.513	0.431
3-61 (Average)	0.688	0.446	0.377
3-75 (Exp)	0.470	0.380	0.340
3-75 (Static)	0.497	0.312	0.260
3-75 (Dynamic)	0.581	0.380	0.314
3-75 (Dynamic+)	0.639	0.437	0.360
3-75 (Average)	0.572	0.376	0.311
33-75 (Exp)	0.610	0.510	0.460
33-75 (Static)	0.476	0.474	0.450
33-75 (Dynamic)	0.574	0.567	0.539
33-75 (Dynamic+)	0.658	0.649	0.617
33-75 (Average)	0.570	0.563	0.535

Supplementary Table 3: **FRET efficiencies for Case study 2: ACTR.** ACTR FRET efficiencies calculated with FRETpredict for all residue pairs (3-61, 3-75, 33-75) at different urea concentrations (0 M, 2.5 M, and 5 M), for the medium rotamer library. The averaging regime is reported in parentheses.

HiSiaP					
Residue pair (conformation)	Exp	Static	Dynamic	Dynamic+	Average
58-134 (open)	0.233	0.232	0.283	0.302	0.272
58-134 (closed)	0.321	0.284	0.364	0.379	0.342
55-175 (open)	0.765	0.554	0.723	0.890	0.722
55-175 (closed)	0.847	0.786	0.942	0.999	0.909
175-228 (open)	0.342	0.210	0.251	0.260	0.240
175-228 (closed)	0.300	0.311	0.388	0.410	0.370
112-175 (open)	0.437	0.260	0.318	0.343	0.307
112-175 (closed)	0.388	0.396	0.486	0.575	0.486
SBD2					
Residue pair (conformation)	Exp	Static	Dynamic	Dynamic+	Average
319-392 (open)	0.408	0.174	0.197	0.215	0.195
319-392 (closed)	0.661	0.614	0.792	0.920	0.775
369-451 (open)	0.275	0.270	0.296	0.304	0.290
369-451 (closed)	0.469	0.477	0.587	0.627	0.564
MalE					
Residue pair (conformation)	Exp	Static	Dynamic	Dynamic+	Average
87-127 (open)	0.740	0.749	0.887	0.959	0.865
87-127 (closed)	0.577	0.515	0.666	0.771	0.651
134-186 (open)	0.903	0.857	0.964	0.994	0.938
134-186 (closed)	0.913	0.819	0.949	0.989	0.919
36-352 (open)	0.401	0.411	0.491	0.530	0.477
36-352 (closed)	0.672	0.548	0.692	0.825	0.688
29-352 (open)	0.219	0.177	0.217	0.237	0.210
29-352 (closed)	0.359	0.321	0.415	0.486	0.407

Supplementary Table 4: **FRET efficiencies for Case study 3: Single structure proteins.** FRET efficiencies calculated with FRETpredict for the open and closed conformations of all the single-structure proteins (HiSiaP, SBD2, and MalE), for the large rotamer library. Every row corresponds to a labeled residue pair, with the protein conformation reported in parentheses. Every column corresponds to an averaging regime or to the experimental value for the specific residue pair and protein conformation.

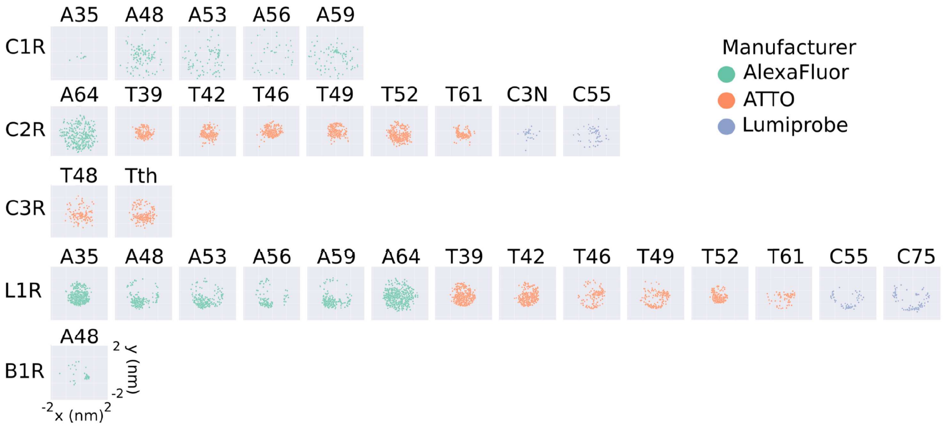

Supplementary Figure 5: **2D projections of the position of the fluorophore's central atom with respect to the Cα atom for the medium rotamer libraries generated in this work.** The projections are obtained as the x and y coordinates of the central atom of the fluorophore (*O91* for AlexaFluor, *C7* for ATTO, and *C10* for Lumiprobe), after placing the Cα atom at the origin. Each plot represents a different FRET probe, divided into rows according to linker type (C1R, C2R, C3R, L1R, B1R, from top to bottom), and colored according to the manufacturer (green for AlexaFluor, orange for ATTO, and blue for Lumiprobe).

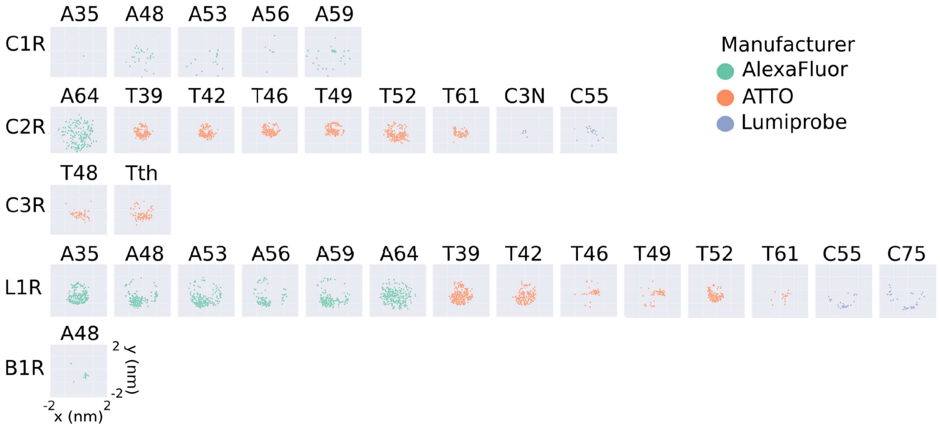

Supplementary Figure 6: **2D projections of the position of the fluorophore's central atom with respect to the Cα atom for the small rotamer libraries generated in this work.** The projections are obtained as the x and y coordinates of the central atom of the fluorophore (*O91* for AlexaFluor, *C7* for ATTO, and *C10* for Lumiprobe), after placing the Cα atom at the origin. Each plot represents a different FRET probe, divided into rows according to linker type (C1R, C2R, C3R, L1R, B1R, from top to bottom), and colored according to the manufacturer (green for AlexaFluor, orange for ATTO, and blue for Lumiprobe).

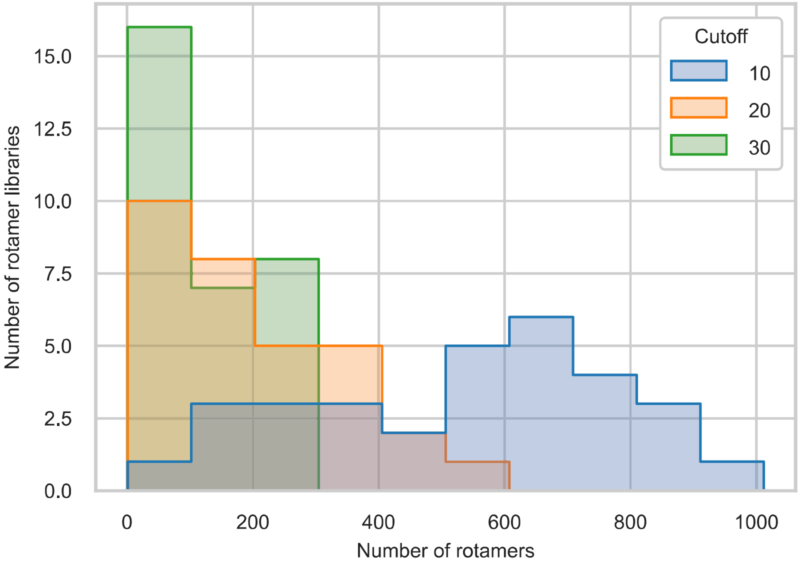

Supplementary Figure 7: **Populations of large, medium, and small rotamer libraries.** Distribution of the number of conformers across all the large (blue), medium (orange), and small (green) rotamer libraries generated in this work. The median number of structures in the large, medium, and small rotamer libraries are 586, 189, and 100, respectively.

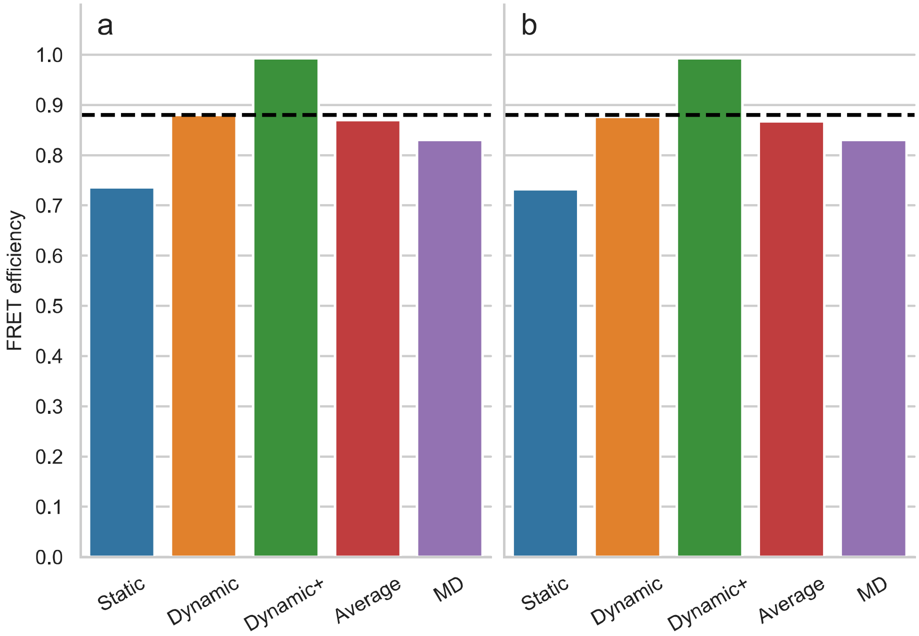

Supplementary Figure 8: **Effect of reweighting on predicted FRET efficiencies (pp11).** FRET efficiency obtained using FRETpredict for the MD trajectory of Polyproline 11 fluorescently labeled at the terminal residues, (**a**) with and (**b**) without reweighting. We calculated E using the large rotamer libraries and for the different regimes (static, dynamic, and dynamic+, in blue, orange, and green, respectively). The graph also shows the average over the three regimes (Average, in red) and the E value obtained from an MD simulation with explicit FRET probes (MD, in purple). The black dashed line indicates the experimental E value.

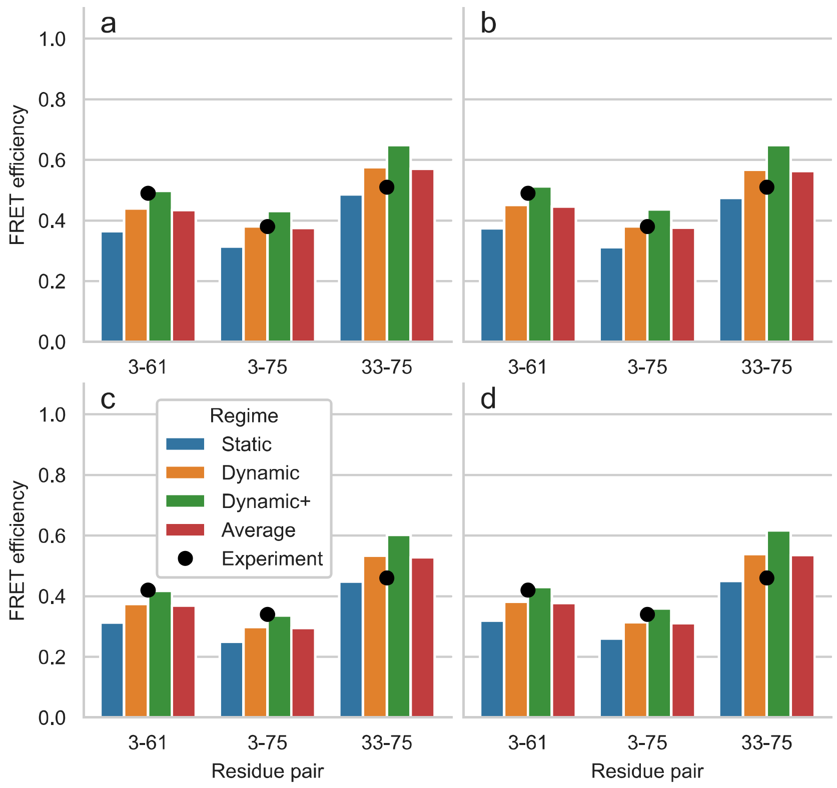

Supplementary Figure 9: **Effect of reweighting on predicted FRET efficiencies (ACTR at [Urea] = 2.5 M and 5 M).** FRET efficiency for ACTR at [urea] = 2.5 M (**a** and **b**), and 5 M (**c** and **d**), with (**a** and **c**) and without (**b** and **d**) reweighting. The protein is fluorescently labeled at three different pairs of sites: 3-61, 3-75, and 33-75. Bars show FRETpredict estimates of the E values calculated using medium rotamer libraries. Predictions for the static, dynamic, and dynamic+ regimes and their average are shown as blue, orange, green, and red bars, respectively. Black circles show the experimental data from Borgia *et al.*[6].

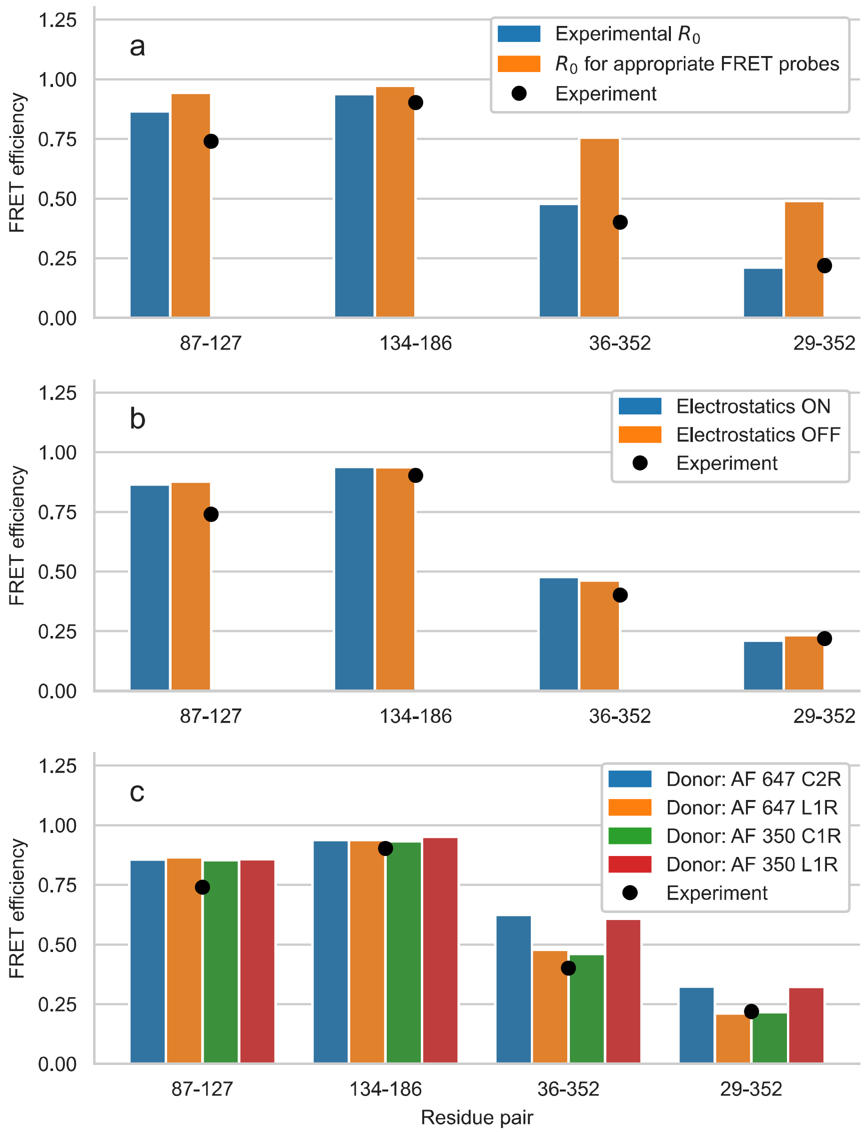

Supplementary Figure 10: **Effect of physicochemical parameters on predicted FRET efficiencies.** Effect of (**a**) R_0, (**b**) electrostatics, and (**c**) probe steric bulk on FRET efficiencies calculated using FRETpredict. Calculations were performed on the open structure of MalE with the large rotamer library. Reported FRET efficiencies in all panels correspond to the average over the different regimes. In panel **a**, the R_0 value is changed from the experimental value of 5.1 nm (blue bars) to the actual R_0 of the two FRET probes used in the calculations (AlexaFluor 647 - AlexaFluor 647), i.e., 6.50 nm (orange bars). In panel **b**, the FRET efficiency was computed by turning electrostatic interactions on (blue bars) or off (orange bars) in the calculation of probe–protein energies. In panel **c**, the donor FRET probe is AlexaFluor 647 C2R (blue bars), AlexaFluor 647 L1R (orange bars), AlexaFluor 350 C1R (green bars), and AlexaFluor 350 L1R (red bars). Black circles show the experimental reference values from Peter *et al.*[7] for each pair of residues.

Supplementary References

[1] Graen, T., Hoefling, M. & Grubmüller, H. AMBER-DYES: Characterization of charge fluctuations and force field parameterization of fluorescent dyes for molecular dynamics simulations. *Journal of Chemical Theory and Computation* **10**, 5505–5512 (2014).

[2] Meng, F. *et al.* Highly disordered amyloid-beta monomer probed by single-molecule FRET and MD simulation. *Biophys. J.* **114**, 870–884 (2018).

[3] Tesei, G. *et al.* DEER-PREdict: Software for efficient calculation of spin-labeling EPR and NMR data from conformational ensembles. *PLOS Computational Biology* **17**, 1–18 (2021).

[4] Polyhach, Y., Bordignon, E. & Jeschke, G. Rotamer libraries of spin labelled cysteines for protein studies. *Phys. Chem. Chem. Phys.* **13**, 2356–2366 (2011).

[5] Huang, J. & MacKerell, A. D., Jr. CHARMM36 all-atom additive protein force field: validation based on comparison to NMR data. *J. Comput. Chem.* **34**, 2135–2145 (2013).

[6] Borgia, A. *et al.* Consistent view of polypeptide chain expansion in chemical denaturants from multiple experimental methods. *Journal of the American Chemical Society* **138**, 11714–11726 (2016).

[7] Peter, M. F. *et al.* Cross-validation of distance measurements in proteins by PELDOR/DEER and single-molecule FRET. *Nature Communications* **13**, 4396 (2022).
